# Chaperones, Canalization, and Evolution of Animal Forms

**DOI:** 10.3390/ijms19103029

**Published:** 2018-10-04

**Authors:** Atsuko Sato

**Affiliations:** 1 Department of Biology, Ochanomizu University, 2-1-1 Otsuka, Bunkyo-ku, Tokyo 112-0012, Japan; pterobranch@gmail.com; Tel.: +81-35978-5377; 2 Marine Biological Association of the UK, The Laboratory, Plymouth PL1 2PB, UK

**Keywords:** maternal inheritance, developmental robustness, bet hedging, epigenetic landscape, DNAJs, heat shock proteins

## Abstract

Over half a century ago, British developmental biologist Conrad Hal Waddington proposed the idea of canalization, that is, homeostasis in development. Since the breakthrough that was made by Rutherford and Lindquist (1998), who proposed a role of Hsp90 in developmental buffering, chaperones have gained much attention in the study of canalization. However, recent studies have revealed that a number of other molecules are also potentially involved in canalization. Here, I introduce the emerging role of DnaJ chaperones in canalization. I also discuss how the expression levels of such buffering molecules can be altered, thereby altering organismal development. Since developmental robustness is maternally inherited in various organisms, I propose that dynamic bet hedging, an increase in within-clutch variation in offspring phenotypes that is caused by unpredictable environmental challenges to the mothers, plays a key role in altering the expression levels of buffering molecules. Investigating dynamic bet hedging at the molecular level and how it impacts upon morphological phenotypes will help our understanding of the molecular mechanisms of canalization and evolutionary processes.

## 1. Robustness and Evolutionary theory

Chaperones have been studied as important players in the maintenance of homeostasis. They are involved from the birth to the death of proteins. When mRNAs are translated, some of the nascent peptides are folded by co-translational folding at the ribosomes, while others are transferred to the endoplasmic reticulum (ER) for correct folding [1]. Upon environmental stress or excess production of nascent peptide chains, some peptides are refolded and they escape from aggregation, while others are delivered to the ER for degradation. None of these processes are conducted without chaperones. Consequently, chaperones have an importance in cellular function impacting on the fitness and lifespan of organisms (reviewed in [2]). For example, the expression of HSF-1, a master transcriptional regulator of heat-inducible gene expression, controls longevity in *Caenorhabditis elegans* [3,4]. Moreover, overexpression of HSF-1 showed a fitness trade-off [5]. In the fruit fly *Drosophila*, a decrease in heat shock protein expression results in a significant decrease in fitness and lifespan [6]. Hormesis, where a low dose of toxic agents bring about beneficial consequences, such as an increase in fitness and lifespan [7,8], might also be a result of increased levels of chaperone expression. Recently, the transgenerational impact of hormesis has been reported [9]. Transgenerational inheritance may not always lead to long-term effects but it can be inherited via transposon insertions [10], suggesting that hormesis might also have evolutionary consequences.

Investigating the role of chaperones in evolutionary processes was inspired by the idea of canalization. In his book ‘The Strategy of the Genes’, Conrad Hal Waddington originally coined the term ‘canalization’ or ‘homeorhesis’, which differs from ‘physiological homeostasis’, referring to some physiological state that is being held constant, and ‘genetic homeostasis’ in which the constant feature of the system is its set of gene frequencies [11] (p.44). He understood the developmental process as a ball falling down a canalized slope in a metaphoric multi-dimensional phase space called the ‘epigenetic landscape’ underpinned by ‘a complex system’ [11]. The idea of canalization has been criticized for not being mathematically defined, thus leading to confusion [12]. However, identifying the importance of canalization was certainly a breakthrough in understanding the evolution of animal forms. More than forty years later, Rutherford and Lindquist dissected the molecular basis of canalization and proposed Hsp90 as a key molecule in canalization [13].

Here, I briefly outline the recent findings that have contributed to our understanding of the role of chaperones in canalization. As an immense amount of work has been published on chaperones in relation to evolution (a topic covered by recent reviews, including [14,15] and references therein), in this review I focus on discussing how studies on the role of chaperones in developmental robustness and evolution can move forward by integration with ecological studies, thus leading to a better understanding of the interaction between the environment and the evolution of animal forms.

## 2. Canalization

**Figure 1:**
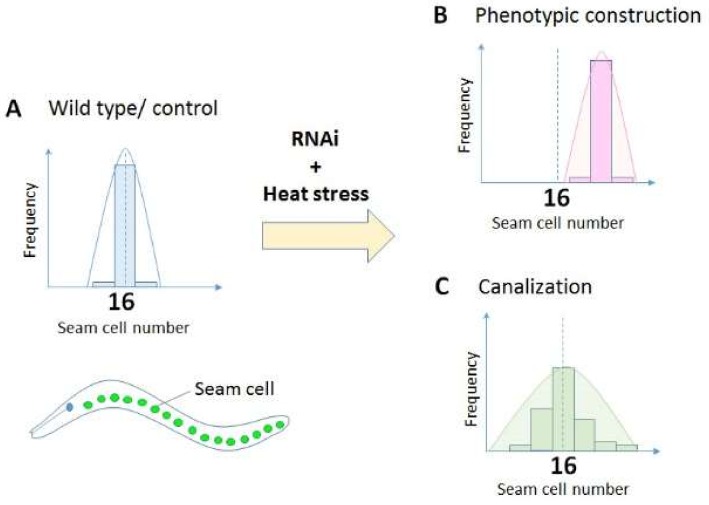
Measurement of canalization using seam cell numbers as a read-out in *Caenorhabditis elegans*. Most wild type worms have 16 seam cells in the lateral side of the body at L4 larval stage (**A**). If a gene is involved in phenotypic construction [18], knockdown of the gene alters the mean seam cell number (**B**). However, if a gene is involved in the canalization of seam cells, knockdown of the gene would increase the variance of seam cell numbers (**C**). See details of the experiments in Section 2.3.

Organismal development is fairly robust in the face of genetic and environmental perturbations. Waddington did not specifically define canalization in a mathematical way; however, he indicated in a figure in his book ‘The Strategy of the Genes’ that ‘canalizing selection’ restrains variation in development under varying environmental conditions across a population [11] (p.66, Figure 9). Canalization can be recognized as a decrease in variance without a shift in the phenotypic mean, which facilitates the ability to distinguish canalization from phenotypic construction, which is defined as control the phenotypic mean (Figure 1) [16,17,18]. Phenotypic plasticity can be confused with developmental robustness or canalization. However, whereas canalization is an insensitivity to genetic or environmental perturbations, plasticity is a reaction of a phenotype against environmental and genetic changes i.e., an opposing effect [19]. Moreover, plasticity includes polyphenisms or morphs, which are a discontinuous range of phenotypes, in addition to reaction norms, a continuous range of phenotypes [20]. Since plasticity is a broad subject and it remains unclear how much its molecular mechanisms are shared with canalization, in this paper, I limit my discussion to canalization and developmental robustness. Plasticity is still an important topic in relation to robustness and therefore I urge readers to refer to previous comprehensive reviews on this topic (such as [21,22]).

There are a number of theoretical considerations on canalization. For example, modelling canalization has predicted that a more robust population can produce greater phenotypic diversity [23,24], which will then be subject to stabilizing selection [25]. Robustness to genetic variation (genetic canalization) and to environmental perturbations (environmental canalization) has been modelled with different inputs [26]. However, environmental canalization and genetic canalization behave similarly to homeostasis [27], indicating that the molecular basis of both genetic canalization and environmental canalization might be shared. Gene network studies have argued that canalization is an inevitable consequence of complex developmental-genetic processes [28]. Genotype networks have, in theory, explained various biological constraints, which can be expanded to genetic constraints on phenotypic variation [29].

### 2.1. Genetic Canalization

The breakthrough that was made by Rutherford and Lindquist (1998) [13] was the demonstration that when the function of Hsp90 is impaired in fruit flies, new phenotypic variants appear. This was followed by similar work in plants [30], bacteria [31] and fish [32,33]. Impairment of Hsp90 function by geldanamycin also led to an increase in destabilizing variants in viral proteins [34]. Overexpression of GroEL [35] and DnaK chaperones [36] increased the number of accumulating mutations in *E*. *coli* over many generations, supporting theoretical predictions (such as [23,24]). Moreover, the impact of synthetic mutations is also buffered by an increased level of expression of chaperones [37].

On the other hand, there is increasing evidence questioning the role of Hsp90 as a ‘capacitor’. For example, studies attempting to identify the role of Hsp90 in phenotypic variation in specific traits of *Drosophila* failed to find convincing evidence of its involvement [38,39,40]. Moreover, the reduction of Hsp90 induced de novo mutations that cause crystalline aggregates in spermatocytes via activation of transposons [41]. This suggested an additional, if not alternative, hypothesis that, instead of buffering pre-existing mutations, a reduction of Hsp90 causes stress-response-like activation and the transposition of transposons, which can cause different insertions depending on the genetic background [41]. In addition, in budding yeast, Hsp90 was found to increase spontaneous mutations and recombinations, suggesting that Hsp90 acts more as a ‘potentiator’ rather than a buffer [42]. Another study showed that cryptic genomic variation does not require reduced robustness [43]. These studies led to another hypothesis that no ‘capacitor’ exists, and that canalization can be explained by ‘genetic modifiers’ that influence the effect of other genes through epistatic interactions [44,45]. In fact, recent studies comparing the effect of gene knockdown in different strains of *C. elegans* showed pronounced differences in effects, suggesting that a genome contains numerous ‘genetic modifiers’ that may have little effect individually but influence penetrance dramatically when combined [46].

The majority of such studies have been on Hsp90, however, there is increasing evidence that there are potentially many molecules that buffer genetic variation. Knockdown analysis for *Ras* signalling pathways has shown that those molecules that are involved in Ras signalling control variability in vulval phenotypes in *C. elegans* [47]. Similarly, the *Hes*-related basic helix-loop-helix transcription factor *lin*-*22* affects the phenotypic variability of the *C. elegans* stem cell-like seam cell numbers, which are usually restricted in number during development [48]. miRNA also masks genetic variants that hinder adult viability [49,50]. Quantitative Trait Loci (QTL) analyses investigating loci interacting with phenotypic traits in *Drosophila* have identified dozens of loci showing trait-specific effects, with four of them interacting in multiple traits [51,52]. Moreover, a genetic screen in *C. elegans* to search for genes interacting with multiple different pathways identified a further six genes that have similar effects to Hsp90 in enhancing the phenotypic consequences of mutations in many different pathways [53]. To describe such molecules, the knockdown of which will affect multiple pathways, Lehner et al. (2006) [53] coined the term ‘hub molecules’. Gene ontology (GO) analysis of ‘hub molecules’ showed that cytoskeletal, DNA damage response, extracellular matrix, splicing, signalling, and transcription factors are all ‘hub molecules’. They did not identify any impact on phenotypic mean or variance by knockdown of ‘hub molecules’, but demonstrated that a number of other genes are potentially involved in canalization. More recently, a large-scale analysis was undertaken to investigate genes that affect variability and means of protein expression when deleted [54]. The resulting data suggested hundreds of genes affecting the variability of protein levels, the function of which spans 50 Gene Ontology categories [54]. Such studies are, however, mostly conducted with laboratory model organisms, which are genetically highly canalized or even isogenic. Heterogeneous populations in the wild might have more complex outcomes in the epigenetic landscape under a single gene manipulation.

### 2.2. Environmental Canalization

Heat shock protein expression has been a major marker of environmental buffering in the study of ecology [55]. However, studies focusing on the molecular basis of environmental canalization have been limited. A study of wild heterogeneous populations of tunicates that are adapted to different thermal environments has shed new light on the molecular basis of environmental canalization [56]. Tunicates are widely accepted as the closest extant group to vertebrates [57]. They are sessile marine organisms that, in the larval stage, exhibit a motile tail and central nervous system, which are similar to tadpole larvae of frogs. The tunicate species *Ciona intestinalis* has been known for hundreds of years since Linnaeus described it from the Atlantic Ocean. *C. intestinalis* has been found in all oceans worldwide. However, recent comparative genomic studies have identified at least two sibling species: type A, which lives in the Atlantic, and type B from the Mediterranean, Japan, and the Pacific coast of America [58,59,60,61,62,63,64]. Comparative studies of the stress response in these species showed that they are adapted to different sea water temperatures [56]. These species can hybridize [64], and cross-hybridization experiments have shown that the level of canalization at higher temperature is maternally inherited [56]. Transcriptomic approaches have revealed that the expression level of *hsp90* is not correlated to maternal inheritance of canalization. Instead, the ER-associated DnaJ chaperones, DNAJC3 and DNAJC10, are important in canalization at warmer temperatures [56].

A caveat of this study is that phenotypic variance and phenotypic mean values were not quantitatively analysed. Also, since the knockdown and knockout methods are still crude in *Ciona*, genetic manipulations cannot easily be performed without changing developmental conditions. To address these issues, Hughes et al. (unpublished) carried out a genetic screening of DnaJ genes in the nematode worm *C. elegans*. In *C. elegans*, RNAi knockdown analysis is routinely achieved by feeding dsRNA to the animal and observing its effect. In addition, the availability of fluorescent GFP reporter transgenic lines permits quantitative analysis of phenotypic variation [65]. Specifically, Hughes et al. exploited a GFP transgenic line that marks the nuclei of neuroepidermal cells called seam cells, which arise in juvenile nematodes and go through a series of re-iterative asymmetric divisions at each larval stage so that the adult animal has two rows of 16 seam cells (Fig 1). By counting seam cell number under various knockdown and temperature conditions, Hughes et al. confirmed that the DNAJC3 orthologue in *C. elegans*, *dnj-7*, increases phenotypic variation, but not the phenotypic mean, at an increased temperature, although exposure to the same temperature does not cause phenotypic variation in wild type worms. In contrast, *dnj-27* knockdown (the human orthologue is DNAJC10) causes phenotypic variation without thermal stress. Moreover, Hughes et al. found additional DnaJ genes that are involved in environmental canalization.

There are at least two important messages stemming from the above observations. One is that some of the DnaJ genes involved in canalization, such as DNAJC10, are involved in protein degradation [66]. Previous global analysis of phenotypic robustness also demonstrated that molecules involved in degradation underpinned genetic robustness in yeast [54]. Computational prediction of protein structures also suggested that disordered proteins are tightly regulated by protein degradation [67]. Together, the evidence suggests that protein degradation can also be important in canalization, although the previous hypothesis proposed that protein folding is key in buffering and thermotolerance [68]. The other important message is that both genetic and environmental canalization can be consequences of ‘a complex system’ that Waddington originally proposed [11,69], i.e., a gene network involving many different genes with various functions, as proposed by Siegal and Bergman (2002) [28]. Like genetic canalization, there are potentially a large number of molecules involved in environmental canalization, rather than just one or two that govern the whole epigenetic landscape. Some of the DnaJs have specific client proteins, whereas others interact with multiple different proteins, i.e., highly interactive proteins [70]. Protein level interactome analysis in combination with gene network analysis will test this hypothesis.

## 3. Importance of Maternal Control in Canalization of Animal Forms

How can robustness be altered via chaperones and impact on evolutionary processes? Rutherford and Lindquist (1998) [13] famously proposed that when a stochastic environment chaperone function is impaired, cryptic phenotypic variation might appear and be subject to natural selection in the evolutionary process. In this article, I argue that it would be more likely that variation in the amount of buffering molecules, rather than stochastic environmental change, alters the whole ‘epigenetic landscape’ that is underpinned by a complex gene network.

Developmental robustness is maternally inherited in many organisms, including sea urchins [71], tunicates [56], and fish [72]. Maternal provision of mRNA and proteins in addition to the maternal genome (including mitochondrial genome) and nutrients can be important cues in controlling the amount of buffering, by altering transcription and translation [73,74]. In addition, epigenetic inheritance of zygotic gene expression can be another important cue. Transgenerational epigenetic inheritance has been a focus of much attention in recent years, and there are a number of review articles that explore its role in evolution [10,75,76,77]. On the other hand, surprisingly, few studies have been carried out on where maternal provision comes from (from maternal cells or the maternal genome in the oocyte) and its impact throughout development. Hence, it has been difficult to dissect the mechanisms of maternal inheritance of canalization.

For example, a number of studies have described the transcriptomic landscape of maternal mRNAs (for example as summarized in [78]), but very few have identified whether these maternal RNAs are from the transcription of the maternal genome in the oocyte or from maternal cells. In *Xenopus*, it has been speculated that the oocyte genome massively transcribes mRNA in the oocyte by forming lampbrush-like chromosomes [79]. However, this speculation was questioned by the observation that massive amplification of mRNA took place before the appearance of lampbrush-like chromosomes [80]. In insects, mRNA and proteins are massively produced by tropic syncytium or nurse cells, and they are transported to oocytes through gap junctions [81,82,83,84]. Such evidence suggests that maternal provision of mRNA to the oocytes may not be limited to insects, yet the molecular details of maternal provision have not been uncovered.

Most maternal mRNAs are degraded during embryonic development when zygotic transcription starts [85]. However, recent studies have revealed the pronounced stability of some maternal mRNA. For example, computational prediction of the degradation pattern of maternal mRNA has shown that some mRNAs, such as ribosomal RNAs, are unexpectedly stable in *Drosophila* embryos [86]. Half-life measurements of maternal mRNAs in yeast have shown that about 1% of mRNAs acting primarily on oxidative phosphorylation were very stable (half-lives > 2h), and that many have polyU sequences in a poly (A) tail [87].

Similarly, studies on the origins and life of maternal proteins are very limited. However, recent mass spectrometry analyses measuring the absolute amount of embryonic proteins in a series of developmental stages of *Xenopus* revealed the importance of maternal proteins during development [88]. The majority of proteins are maternally supplied and only 9% of all embryonic proteins could be synthesized in a 24-h period [88], which is consistent with previous studies showing little new protein synthesis from fertilization to neurulation [89]. Whereas transcription factors and tissue specific proteins are translated on demand, highly abundant proteins, typically metabolic enzymes, do not change in abundance during development. The authors concluded that the majority of maternally loaded proteins remain during the entire developmental process, but how activation and inactivation of these maternal proteins is controlled remains unknown [88].

**Figure 2:**
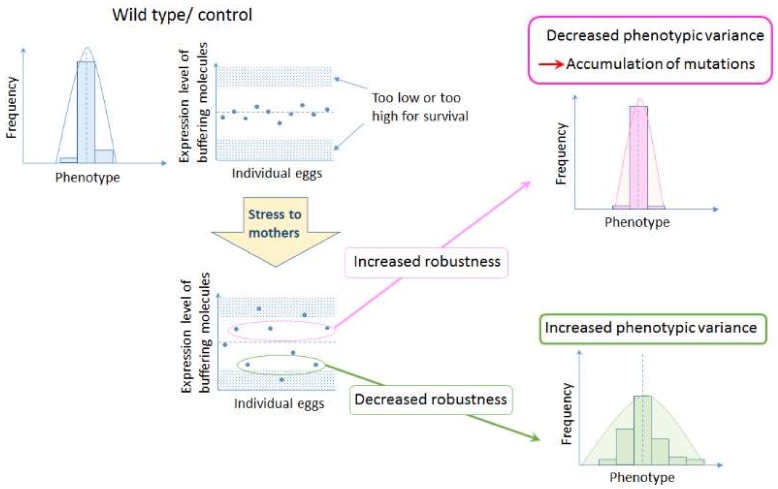
Hypothesis on the role of bet hedging in altering developmental robustness that leads to evolution of organismal forms. Increased variation by maternal environment controls expression levels of buffering molecules such as chaperones. If the level of expression increases, developmental robustness will increase, allowing the accumulation of background mutations. On the other hand, if the level of expression decreases, developmental robustness will decrease, increasing the susceptibility to genetic variations and all the epistatic interactions involved, resulting in a change in the ‘epigenetic landscape’.

A number of ecological studies have shown that maternal environment affects offspring size (see summary of some examples in [90]). The phenomenon of ‘dynamic (or diversification) bet hedging’, as proposed by Crean and Marshall (2009) i.e., variation in offspring phenotypes induced by unpredictable environmental challenges [91,92], has been observed from bacteria [93] to terrestrial invertebrates [94] and marine invertebrates [90]. The observed variation in offspring size might be a consequence of maternal provision of mRNA and proteins, yet the molecular mechanisms that underpin this are still largely uninvestigated. I propose that dynamic bet hedging controls canalization levels in offspring by increasing the variation in the amount of buffering molecules, such as chaperones when mothers are exposed to unpredictable environmental challenges (Figure 2). An increase or decrease in the amount of gene expression can alter a complex gene network, and all the epistatic interactions involved, resulting in an alteration of the whole ‘epigenetic landscape’.

Environmental stress induces up and down-regulation of many genes, not just those that are related to stress response [95]. Previous studies have identified examples of bet hedging at the molecular level in bacteria and yeast [93,96], yet transgenerational inheritance of bet hedging has not yet been empirically described [76,77]. However, experimental manipulation of maternal provision has shown the importance of maternal control in the molecular basis of a stress response and its transgenerational inheritance. For example, genetic manipulation of *Hsp23* expression level in *Drosophila* ovaries resulted in embryos and adults with decreased thermal susceptibility during development [97]. Furthermore, maternally inherited *Polycomb* levels reduced the response to toxic stress in fruit flies, which can be inherited over several generations [98]. These studies indicate that maternal provision impacts on offspring developmental robustness, the effect of which can be inherited transgenerationally. Testing whether the maternal environment impacts upon the maternal provision of buffering molecules and consequently on phenotypic variation will be an important focus of future studies.

## 4. Conclusions and Perspectives

Amongst various hypotheses on the mechanisms of ‘developmental buffering’ that Waddington proposed, Hsp90 have been paid the most attention. However, recent studies have revealed a wide range of molecules that are involved in canalization, including DnaJ chaperones. Now, the key is to understand how the environment alters the expression levels of such molecules leading to alterations in the epigenetic landscape, and how these changes can be fixed in a population. Given that developmental robustness is maternally inherited in various organisms, more attention should be given to understanding the molecular basis of maternal control of developmental buffering, through maternal provision of buffering molecules. Furthermore, it would be intriguing to test whether the environment of the mother can alter the levels of buffering molecules maternally provided to the oocytes, and how this impacts on morphological phenotypes. Exploring the mechanisms of the maternal inheritance of canalization will contribute to our understanding of the evolutionary process over the past 500 million years of evolutionary history.

## Abbreviations

HSP	Heat shock protein
GFP	Green fluorescent protein
